# Supplementary material for: Amphiphile-CpG vaccination induces potent lymph node activation and COVID-19 immunity in mice and non-human primates
Source: NPJ Vaccines. 2022 Oct 28;7:128. doi: 10.1038/s41541-022-00560-3 (PMC9616425; doi:10.1038/s41541-022-00560-3)
Supplement: Supplementary file 1 — Supplemental Material [file 41541_2022_560_MOESM1_ESM.pdf]

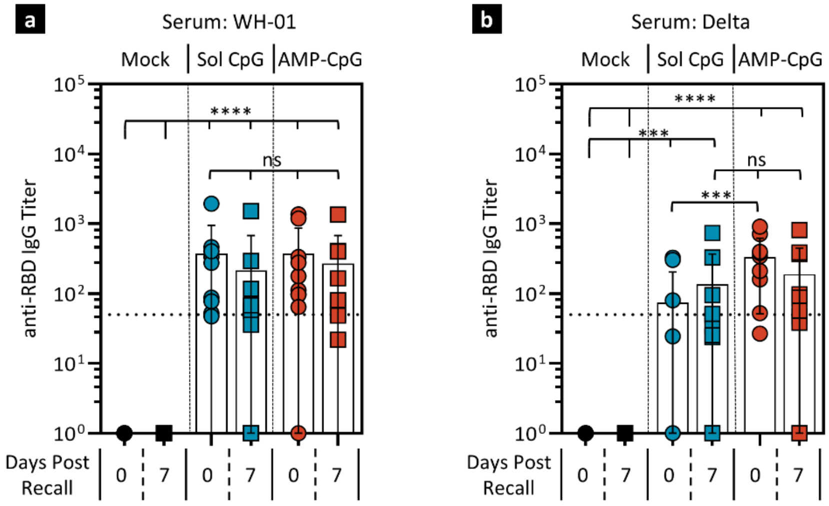

Supplementary Fig. 1 Serum antibody responses induced following SARS-CoV-2 Spike RBD immunization in mice.

C57Bl/6J mice (n = 10) were immunized twice with 10 µg WH-01 RBD protein and 1 nmol soluble or AMP-CpG. 30 weeks post dose 2, mice were challenged subcutaneously with 10 µg WH-01 RBD protein. **a-b** Blood serum titers of anti-SARS-CoV-2 RBD antibodies were assayed 1 day before and 7 days after antigen challenge for WH-01 (**a**) and Delta (**b**). Values depicted are means ± standard deviation. *ns, not significant, ****p < 0.0001* by one-way ANOVA followed by Tukey's post-hoc analysis applied to antibody titers.

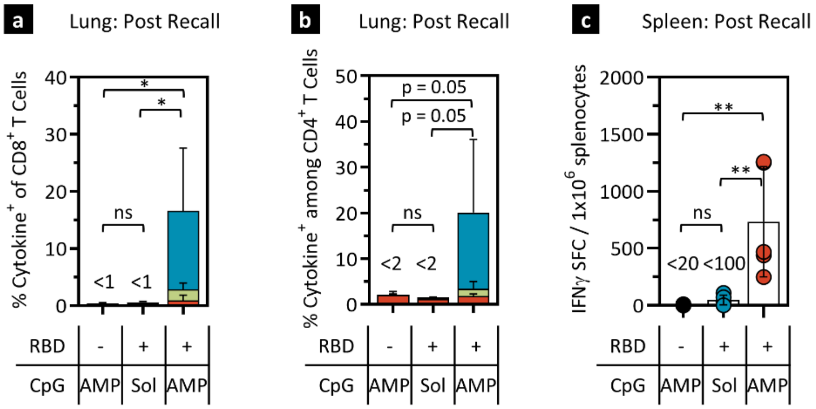

Supplementary Fig. 2 Intranasal antigen exposure recalls T cell responses generated by immunization with AMP-CpG in mice.

C57Bl/6J mice (n=5) were immunized twice with 10 µg WH-01 RBD protein and 1 nmol soluble or AMP-CpG. **a-c** 30 weeks post dose 2, mice were challenged intranasally with 10 µg WH-01 RBD protein and assayed 7 days later. $CD8^+$ **(a)** and $CD4^+$ **(b)** T cells collected from perfused lung were stimulated with WH-01 RBD OLPs and assayed for intracellular cytokines by flow cytometry. **c** Splenocytes were restimulated with WH-01 RBD OLPs and assayed for IFNγ production by ELISpot. Mock vaccines contained AMP-CpG without the addition of antigen. Values depicted are means ± standard deviation. **p < 0.05, **p < 0.01* by one-way ANOVA followed by Tukey's post-hoc analysis applied to cytokine+ T-cell frequencies, or SFC numbers.

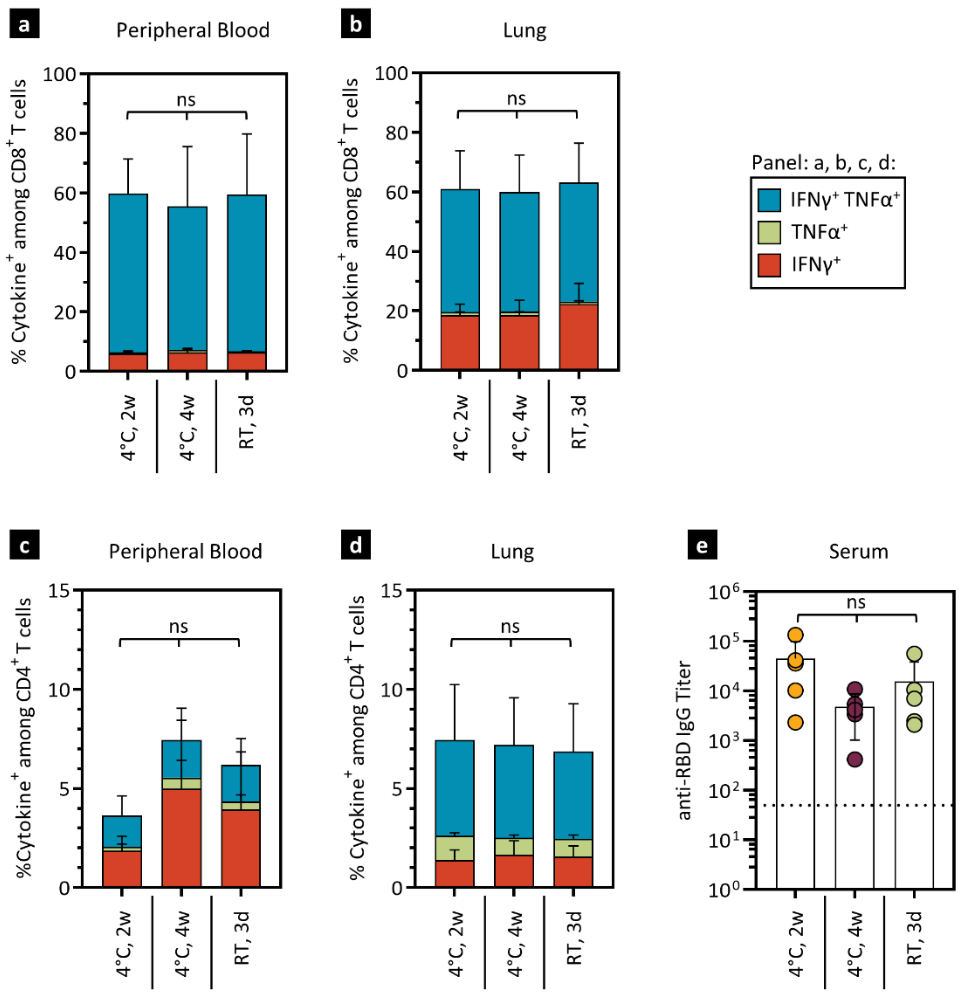

Supplementary Fig. 3 ELI-005 retains immunogenicity upon storage at refrigerated or ambient temperatures.

ELI-005 vaccine doses, consisting of 10 µg WH-01 RBD protein and 1 nmol AMP-CpG, were admixed and stored at 4°C or 22°C (RT, room temperature) for the indicated period. C57Bl/6J mice (n=5) were immunized twice at week 0 and 2 and assayed 7 days after. $CD8^+$ T cells from peripheral blood (**a**) and perfused lung (**b**), as well as $CD4^+$ T cells (**c-d**) from those tissues were stimulated overnight with overlapping WH-01 RBD peptides and assayed for intracellular cytokines by flow cytometry. **e** 7 days post booster dose, blood serum was analyzed for anti-SARS-CoV-2 RBD antibody titers against WH-01 RBD. *ns,*

not significant, by one-way ANOVA followed by Tukey's post-hoc analysis applied to T cell frequencies and antibody titers.

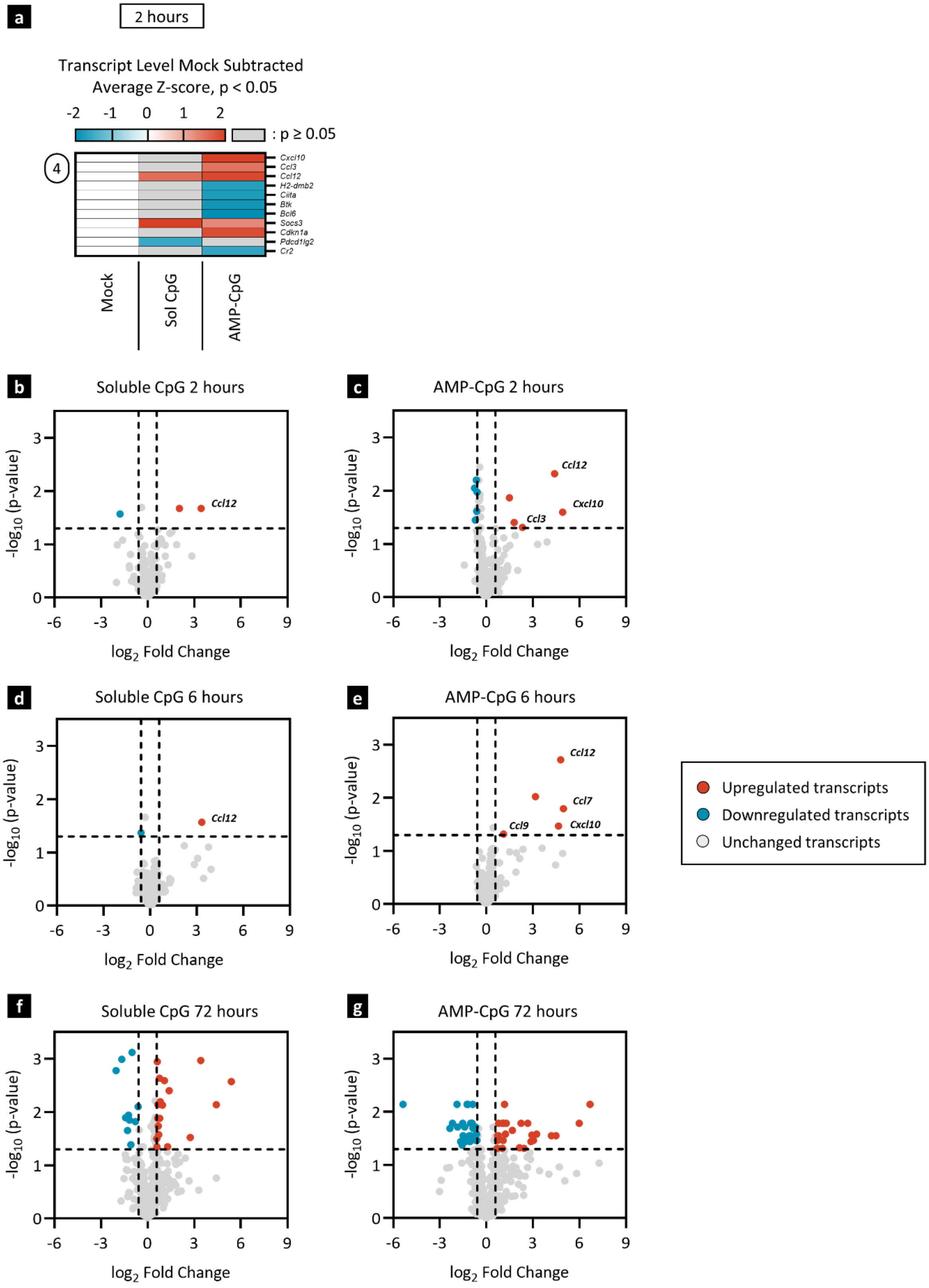

a
2 hours
Transcript Level Mock Subtracted
Average Z-score, p < 0.05
-2 -1 0 1 2
: p ≥ 0.05
4
Cxcl10
Ccl3
Ccl12
H2-dmb2
Ciita
Btk
Bcl6
Socs3
Cdkn1a
Pdcd1lg2
Cr2
Mock
Sol CpG
AMP-CpG
b
Soluble CpG 2 hours
c
AMP-CpG 2 hours
Ccl12
Cxcl10
Ccl3
d
Soluble CpG 6 hours
e
AMP-CpG 6 hours
Ccl7
Ccl9
f
Soluble CpG 72 hours
g
AMP-CpG 72 hours
-log10 (p-value)
log2 Fold Change
Upregulated transcripts
Downregulated transcripts
Unchanged transcripts

Supplementary Fig. 4 AMP-CpG immunization induces lymph node transcriptional reprogramming reflecting APC recruitment with increased potential for antigen processing and presentation.

a Heatmap representation of whole lymph node mRNA analyzed by NanoString nCounter® Mouse Immunology Panel. Shown are mock-subtracted, average Z-scores of gene transcript levels significantly ($p < 0.05$) downregulated (≥ -1.5-fold change, blue) or upregulated (≥ 1.5-fold change, red) at 2 hours post injection relative to mock immunization. Insignificant values with $p \geq 0.05$ are shown in gray. Gene groups follow the same numbering scheme as in **fig 3**. **b-g** Volcano plot representation of log-transformed soluble CpG and AMP-CpG transcript values at 2 hours (**b-c**), 6 hours (**d-e**), and 72 hours (**f-g**) post injection representing data from **supplementary figure 3a**, and figure **3b** and **3f**, respectively. Mock vaccines contained PBS vehicle only. Dotted horizontal line represents significance threshold of $p = 0.05$; vertical dotted lines represent fold-change limits of ± 1.5-fold change. Statistical analysis was performed using Rosalind software.

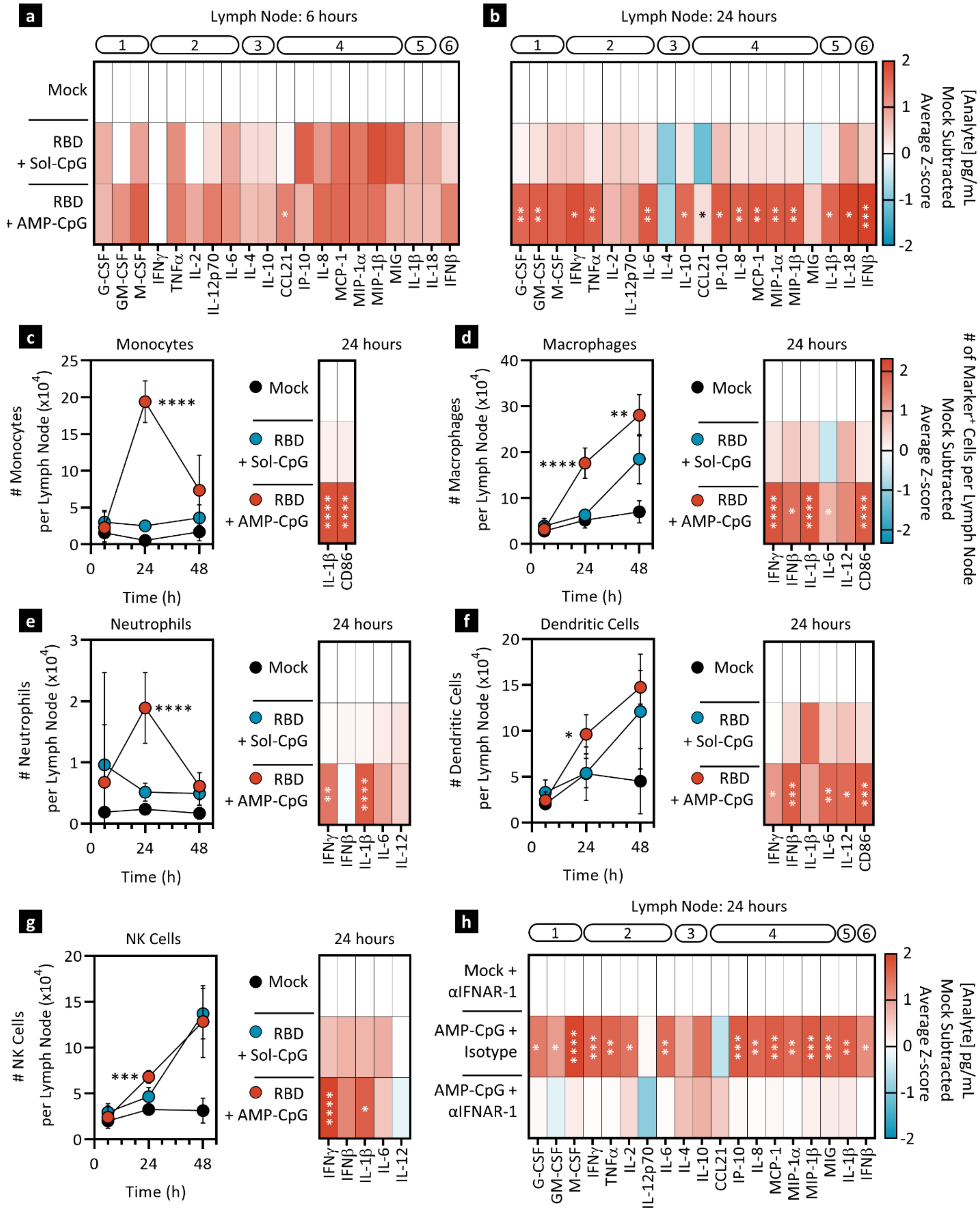

a
Lymph Node: 6 hours
1
2
3
4
5
6
Mock
RBD + Sol-CpG
RBD + AMP-CpG
G-CSF
GM-CSF
M-CSF
IFNγ
TNFα
IL-2
IL-12p70
IL-6
IL-4
IL-10
CCL21
IP-10
IL-8
MCP-1
MIP-1α
MIP-1β
MIG
IL-1β
IL-18
IFNβ
b
Lymph Node: 24 hours
[Analyte] pg/mL Mock Subtracted Average Z-score
c
Monocytes
Monocytes per Lymph Node (x10^4)
Time (h)
24 hours
Mock
IL-1β
CD86
d
Macrophages
Macrophages per Lymph Node (x10^4)
of Marker⁺ Cells per Lymph Node Mock Subtracted Average Z-score
e
Neutrophils
Neutrophils per Lymph Node (x10^4)
f
Dendritic Cells
Dendritic Cells per Lymph Node (x10^4)
g
NK Cells
NK Cells per Lymph Node (x10^4)
h
Mock + αIFNAR-1
AMP-CpG + Isotype
AMP-CpG + αIFNAR-1

Supplementary Figure 5. Additional statistical analysis for fig 4.

Data reported in **fig 4** were re-analyzed to determine statistical significance between groups immunized with soluble and AMP-CpG, whereas statistical analysis in fig 4 compared to mock treatment. Values depicted are mean ± standard deviation. *$p < 0.05$; **$p < 0.01$; ***$p < 0.001$; ****$p < 0.0001$ by one-way ANOVA followed by Šidák's post-hoc analysis comparing immunization with soluble CpG to AMP-CpG.

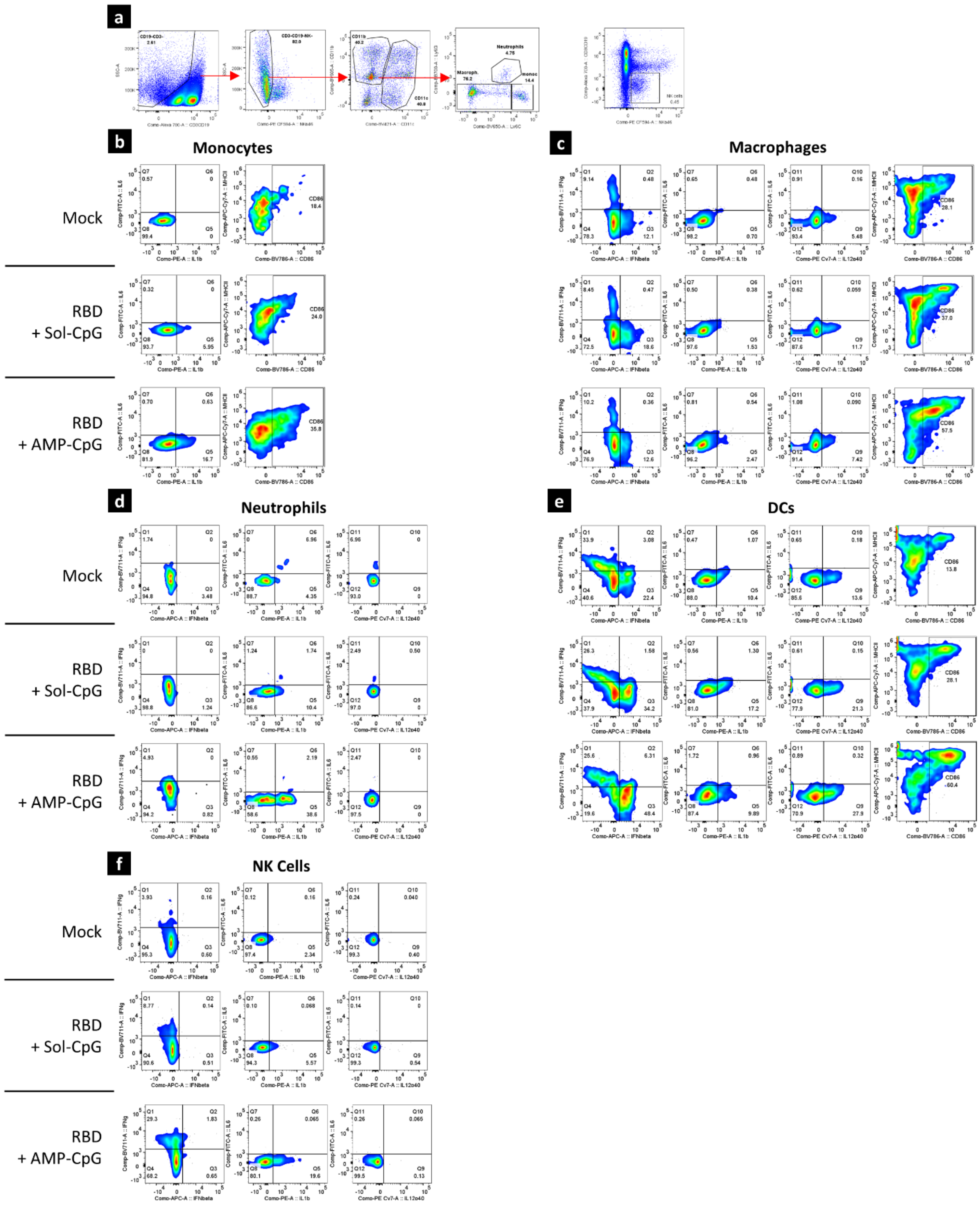

Supplementary Fig. 6. Supplementary data for fig 4.

Flow cytometric analysis strategy and example scatter plots for surface marker and intra-cellular cytokine expression induced in innate immune cell lineages.

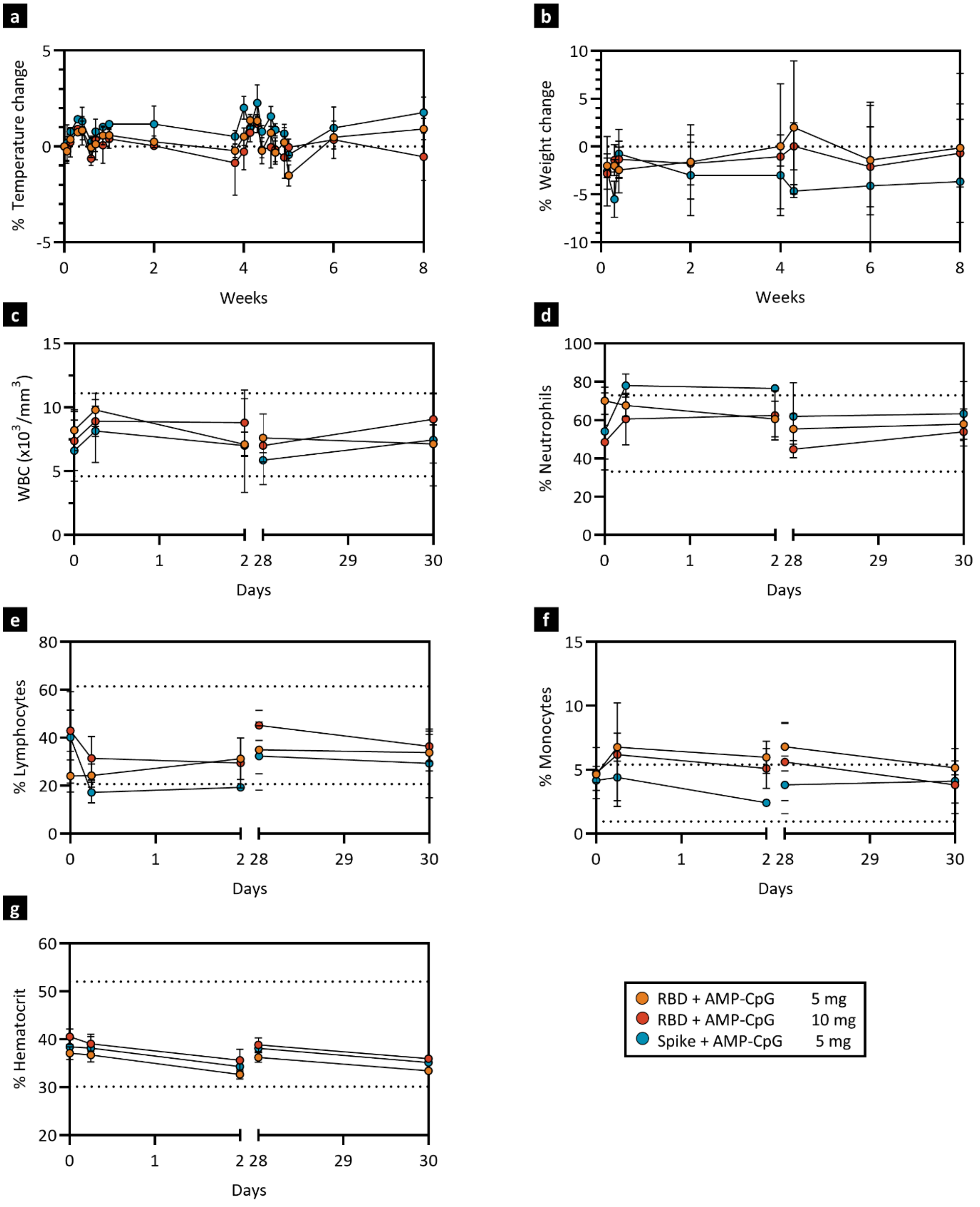
a
% Temperature change
Weeks
b
% Weight change
Weeks
c
WBC (x10^3/mm^3)
Days
d
% Neutrophils
Days
e
% Lymphocytes
Days
f
% Monocytes
Days
g
% Hematocrit
Days
RBD + AMP-CpG 5 mg
RBD + AMP-CpG 10 mg
Spike + AMP-CpG 5 mg

Supplementary Fig. 7 ELI-005 vaccination is safe and non-toxic in NHP.

Rhesus macaques (n=2/3) were immunized at week 0 and 4 with 140 µg WH-01 RBD protein admixed with either 5 mg or 10 mg of AMP-CpG, or 140 µg full WH-01 Spike protein admixed with AMP-CpG at 5 mg. Vital signs were recorded. Shown are percent change for temperature (**a**) and weight change (**b**) from baseline. Sera was collected at multiple timepoints for assessment in a hematology complete blood count panel. Shown are white blood cells (WBC) (**c**), % neutrophils (**d**), % lymphocytes (**e**), % monocytes (**f**), and % hematocrit (**g**). Dotted lines indicate reference value ranges.
