# Supplementary material for: Amphiphile-CpG vaccination induces potent lymph node activation and COVID-19 immunity in mice and non-human primates
Source: NPJ Vaccines. 2022 Oct 28;7:128. doi: 10.1038/s41541-022-00560-3 (PMC9616425; doi:10.1038/s41541-022-00560-3)
Supplement: Supplementary file 2 — REPORTING SUMMARY [file 41541_2022_560_MOESM2_ESM.pdf]

naturereseach

Corresponding author(s): Dr Peter DeMuth

Reporting Summary

Nature Research wishes to improve the reproducibility of the work that we publish. This form provides structure for consistency and transparency in reporting. For further information on Nature Research policies, see Authors & Referees and the Editorial Policy Checklist.

Statistical parameters

When statistical analyses are reported, confirm that the following items are present in the relevant location (e.g. figure legend, table legend, main text, or Methods section).

n/a	Confirmed	
☐	☒	The exact sample size (n) for each experimental group/condition, given as a discrete number and unit of measurement
☐	☒	An indication of whether measurements were taken from distinct samples or whether the same sample was measured repeatedly
☐	☒	The statistical test(s) used AND whether they are one- or two-sided<br>*Only common tests should be described solely by name; describe more complex techniques in the Methods section.*
☐	☒	A description of all covariates tested
☒	☐	A description of any assumptions or corrections, such as tests of normality and adjustment for multiple comparisons
☐	☒	A full description of the statistics including central tendency (e.g. means) or other basic estimates (e.g. regression coefficient) AND variation (e.g. standard deviation) or associated estimates of uncertainty (e.g. confidence intervals)
☐	☒	For null hypothesis testing, the test statistic (e.g. F, t, r) with confidence intervals, effect sizes, degrees of freedom and P value noted<br>*Give P values as exact values whenever suitable.*
☒	☐	For Bayesian analysis, information on the choice of priors and Markov chain Monte Carlo settings
☒	☐	For hierarchical and complex designs, identification of the appropriate level for tests and full reporting of outcomes
☒	☐	Estimates of effect sizes (e.g. Cohen's d, Pearson's r), indicating how they were calculated
☐	☒	Clearly defined error bars<br>*State explicitly what error bars represent (e.g. SD, SE, CI)*

Our web collection on statistics for biologists may be useful.

Software and code

Policy information about availability of computer code

Data collection	Flow cytometric data acquisition was performed on BD FACS Symphony or BD FACSCanto II and data were analyzed with BD FlowJo V10 software.
Data analysis	Flow cytometric data acquisition was performed on BD FACS Symphony or BD FACSCanto II and data were analyzed with BD FlowJo V10 software. Transcriptional responses were assessed with nSolver software v4.0 (NanoString Technologies) and differential gene expression was carried out using ROSALIND software. Statistical analysis was performed using Graphpad Prism v9.4.

For manuscripts utilizing custom algorithms or software that are central to the research but not yet described in published literature, software must be made available to editors/reviewers upon request. We strongly encourage code deposition in a community repository (e.g. GitHub). See the Nature Research guidelines for submitting code & software for further information.

Data

Policy information about availability of data

All manuscripts must include a data availability statement. This statement should provide the following information, where applicable:

- Accession codes, unique identifiers, or web links for publicly available datasets
- A list of figures that have associated raw data
- A description of any restrictions on data availability

The datasets supporting the findings presented in this study are available from the corresponding author upon reasonable request. All requests for data will be promptly reviewed by Elicio Therapeutics to verify whether the request is subject to any intellectual property obligations. Any data that can be shared will be released via a Material Transfer Agreement

Field-specific reporting

Please select the best fit for your research. If you are not sure, read the appropriate sections before making your selection.

☒ Life sciences ☐ Behavioural & social sciences

For a reference copy of the document with all sections, see nature.com/authors/policies/ReportingSummary-flat.pdf

Life sciences

Study design

All studies must disclose on these points even when the disclosure is negative.

Sample size	This is not a human clinical trial. No formal sample size calculations were performed as appropriate for preclinical evaluation. 5-10 animals per group which is standard for the field were used to provide sufficient statistical power to support analysis based on prior studies of immunogenicity assessment.
Data exclusions	No data were excluded from the analysis.
Replication	All attempts at replication were successful.
Randomization	Mice and NHPs were random allocated into each experimental group.
Blinding	Investigators were not blinded.

Materials & experimental systems

Policy information about availability of materials

n/a	Involved in the study
☒	☐ Unique materials
☐	☒ Antibodies
☒	☐ Eukaryotic cell lines
☐	☒ Research animals
☒	☐ Human research participants

Antibodies

Antibodies used

Live/Dead fixable stain (Aqua, Invitrogen, cat# L34966)
CD11b (BV605, clone: M1/70, BioLegend)
CD11c (BV421, clone: N418, BioLegend)
CD3 (AF700, clone: 17A2, BioLegend)
CD19 (AF700, clone: 6D5, BioLegend)
Ly6C (BV650, clone: HK1.4, BioLegend)
Ly6G (PcP-Cy5.5, clone: 1A8, BioLegend)
NKp46 (PE-Dazzle594, clone: 29A1.4, BioLegend)
MHCII (APC-Cy7, clone: M5/114.15.2, BioLegend)
CD86 (BV785, clone: GL-1, BioLegend)
IFNγ (BV711, clone: XMG1.2, BioLegend)
IL12p40 (PECy7, clone: C15.6, BioLegend)
IL1β (PE , clone: NJTEN3, Invitrogen)
IL6 (AF488, clone: MP5-20F3, Invitrogen)
IFNβ (APC, ASSAYPRO, cat# 32183-05161T)
CD4 (PE-Cy5.5, clone: S3.5, Invitrogen)

CD8 (AF647, clone: RPA-T8, BioLegend)
CD45RA (FITC, clone: 5H9, BD)
CCR7 (BV650, clone: G043H7, BioLegend)
aqua live/dead dye (Invitrogen, L34957)
CD3 (APC-Cy7, clone: SP34-2, BD)
CD69 (ECD, clone: TP1.55.3, Beckman Coulter)
IFNγ (AF700, clone: B27, BioLegend)
IL-2 (BV421, clone: MQ1-17H12, BioLegend)
IL-4 (PE, clone: 8D4-8, BioLegend)
TNFα (BV605, clone: MAb11, BioLegend)
IL-17A (PE-Cy7, clone: BL168, BioLegend)
RBD-PE tetramer VNFNFNGL (NIH Tetramer Core Facility at Emory University, cat# 54971)
CD8a (APC, clone: 53-6.7, eBioscience)
CD3 (APC-Cy7, clone: 17A2, BD)
CD44 (PE-Cy7, clone: IM7, eBioscience)
CD62L (FITC, clone: MEL-14, eBioscience)
Live/Dead fixable (aqua) dead cell stain kit (Invitrogen, cat# L34966)
aqua live/dead dye (Invitrogen, L34957)
anti-human IgM (FITC, clone: G20-127, BD)
anti-human IgG (PE-Cy7, clone: G18-145, BD)
anti-human CD3 (AF700, clone: SP34-2, BD)
anti-human PD-1 (BV650, clone: EH12.1, BD)
anti-human CD20 (PE/Dazzle 594, clone: 2H7, BioLegend)
anti-human CD4 (APC-Cy7, clone: OKT4, BioLegend)
anti-human CXCR5 (PcP-eF710, clone: MU5UBEE, ThermoFisher
anti-human Bcl-6 (PE, clone: 7D1, BioLegend)
anti-human Ki-67 (BV421, clone: 11F6, BioLegend)

Validation

Validation was based on the manufacturer's website.

Research animals

Policy information about studies involving animals; ARRIVE guidelines recommended for reporting animal research

Animals/animal-derived materials

For mouse studies, female 6- to 8-week-old C57BL/6J mice were purchased from the Jackson Laboratory (Bar Harbor, ME). For NHP studies, 8 outbred, Indian-origin, 4-5 year old female rhesus macaques (Macaca mulatta) were provided by New Iberia Research Center (New Iberia, LA).

Method-specific reporting

n/a	Involved in the study
☒	☐ ChIP-seq
☐	☒ Flow cytometry
☒	☐ Magnetic resonance imaging

Flow Cytometry

Plots

Confirm that:

☒ The axis labels state the marker and fluorochrome used (e.g. CD4-FITC).

☒ The axis scales are clearly visible. Include numbers along axes only for bottom left plot of group (a 'group' is an analysis of identical markers).

☒ All plots are contour plots with outliers or pseudocolor plots.

☒ A numerical value for number of cells or percentage (with statistics) is provided.

Methodology

Sample preparation

Mouse peripheral blood cells were collected 7 days after each booster dose and lung-resident leukocytes were collected after the final booster dose, using retro-orbital bleeding techniques. Blood was collected through Heparin coated capillary tubes (14705-003, VWR) into Blood Collection Tubes (22-030-403, Fisher Scientific). Blood samples were lysed using a 1:10 volumetric ratio of blood to ACK Lysis Buffer (50-983-219, Fisher Scientific) for 5min on ice. Lysis buffer was neutralized with a 1:3 volumetric ratio of lysed blood to PBS, and samples were centrifuged at 300xG for 5min. Samples were subsequently washed with PBS and resuspend in complete media. Lungs were harvested following perfusion with 10 mL of PBS into the right ventricle of the heart. Lung tissue was physically dissociated and digested with RMPI1640 media containing 1 mg/mL collagenase D and 25 units/mL DNaseI.
For NHP studies, frozen PBMCs were thawed and rested overnight. 106 PBMCs/well were resuspended in R10 media supplemented with anti-CD49d monoclonal antibody (clone: 9F10, BD), anti-CD28 monoclonal antibody (clone: CD28.2, BD), and Golgi inhibitors monensin (Fisher Scientific, cat# NC0176671) and brefeldin A (Fisher Scientific, cat# 50-112-9757) and incubated at 37°C for 8 hours, then maintained at 4°C overnight. The next day, cells were surface-stained with antibodies against CD4 (PE-

Cy5.5, clone: S3.5, Invitrogen), CD8 (AF647, clone: RPA-T8, BioLegend), CD45RA (FITC, clone: 5H9, BD), CCR7 (BV650, clone: G043H7, BioLegend), and aqua live/dead dye (Invitrogen, L34957), and subsequently fixed with BD CytoFix/CytoPerm (BD, 554714). Cells were further stained with antibodies against CD3 (APC-Cy7, clone: SP34-2, BD), CD69 (ECD, clone: TP1.55.3, Beckman Coulter), IFNγ (AF700, clone: B27, BioLegend), IL-2 (BV421, clone: MQ1-17H12, BioLegend), IL-4 (PE, clone: 8D4-8, BioLegend), TNFα (BV605, clone: MAb11, BioLegend), and IL-17A (PE-Cy7, clone: BL168, BioLegend). Cells fixed in 1.5% formaldehyde were acquired on a BD FACS Symphony and data were analyzed with BD FlowJo V10 software.

Instrument	Flow cytometric data acquisition was performed on BD FACS Symphony or BD FACSCanto II and data were analyzed with BD FlowJo V10 software.
Software	Flow cytometric data acquisition was performed on BD FACS Symphony or BD FACSCanto II and data were analyzed with BD FlowJo V10 software.
Cell population abundance	NA
Gating strategy	For mouse samples, the gating strategy used for intracellular cytokine stained (ICS) flow cytometry on blood and perfused lung samples was to first gate on lymphocytes by FSC/SSC, then singlets and then live cells. Next CD3+ cells were gated and from that population CD4+ and CD8+ T-cells were determined. IFNy and TNFa were gated from each of the CD4+ and CD8+ T-cell populations. For MHC-tetramer and memory phenotype staining lymphocytes were gated by FSC/SSC, then singlets and then live cells. Next CD3+ cells were gated and from that population CD8+ T-cells were determined. Tetramer+ cells were gated from the CD8+ population. Further, CD44+/CD62L+ were gated from the tetramer+ cells.<br><br>For activation marker and ICS assessment of mouse lymph node, samples were first gated on live cells, then singlets, then lymphocytes. Next CD3-CD19- cells were gated and from that population, the NK+ and NK- cells were determined by gating on NKp46. The NKp46- gate was the parent population for CD11c+ DC and for CD11b+ myeloid cells. From this parent, the CD11b+ gate was used to determine lymph node resident Macrophages and infiltrating monocytes and Neutrophils. CD86 and various cytokines were gated from each cell subset, relative to the Mock group.<br>For NHP samples, the gating strategy used for ICS was to first gate on live cells, then singlets and then lymphocytes. Next CD3+ cells were gated and from that population CD4+ and CD8+ T-cells were determined. The cytokines were gated individually against the activation marker CD69 from each of the CD4+ and CD8+ T-cell populations. The gating strategy used for GC B cells was to first gate on live cells, then singlets and then lymphocytes. Next CD19+ B cells were gated and from that population Ki67+ Bcl6+ germinal center cells were determined. The RBD tetramer+ B cells were gated from the germinal center cell population.

☒ Tick this box to confirm that a figure exemplifying the gating strategy is provided in the Supplementary Information.
